# Outcomes associated with comorbid diabetes among patients with COPD exacerbation: findings from the ACURE registry

**DOI:** 10.1186/s12931-020-01607-6

**Published:** 2021-01-06

**Authors:** Xihua Mao, Chen Liang, Hongtao Niu, Fen Dong, Ke Huang, Yahong Chen, Kewu Huang, Qingyuan Zhan, Yaowen Zhang, Yin Huang, Ting Yang, Chen Wang

**Affiliations:** 1 Chinese Alliance for Respiratory Diseases in Primary Care, Beijing, China; 2 Department of Pulmonary and Critical Care Medicine, Center of Respiratory Medicine, China-Japan Friendship Hospital, No 2, East Yinghua Road, Chaoyang District, Beijing, 100029 China; 3 National Clinical Research Center for Respiratory Diseases, Beijing, China; 4 Institute of Respiratory Medicine, Chinese Academy of Medical Science, Beijing, China; 5 Institute of Clinical Medical Sciences, China-Japan Friendship Hospital, Beijing, China; 6 Department of Pulmonary and Critical Care Medicine, Peking University Third Hospital, Beijing, China; 7 Department of Pulmonary and Critical Care Medicine, Beijing Chao-Yang Hospital, Beijing, China; 8 Department of Respiratory Medicine, Capital Medical University, Beijing, China; 9 Chinese Academy of Medical Sciences and Peking Union Medical College, 9 Dongdan 3rd Alley, Dong Dan, Dongcheng, Beijing, 100730 China

**Keywords:** Chronic obstructive pulmonary disease, Exacerbation, Diabetes, Clinic outcomes, Cost

## Abstract

**Background:**

Diabetes is a common comorbidity among patients with exacerbation of chronic obstructive pulmonary disease (AECOPD). Diabetes has been reported to be associated with length of stay (LOS), death, and cost among AECOPD patients. However, most studies are retrospective or have small sample sizes. The association for cost has not been researched using diabetes as a main analytic factor. This study aimed to fill gaps mentioned above, to compare basic characteristics between the diabetic and non-diabetic group, and to detect associations between diabetes and clinical outcomes among patients hospitalized with AECOPD.

**Methods:**

A total of 5334 AECOPD patients, classified into diabetic and non-diabetic group, were included from a prospective multicenter patient registry study. Generalized linear regression and logistic regression were separately used for the association between diabetes and direct hospitalization cost and the association between diabetes and LOS.

**Results:**

Generally, diabetic patients had a more severe profile, including being older, more overweight or obese, having more former smokers, more emergency room visits in the past 12 months, and more comorbidities occurrence. Diabetic patients also had worse clinical outcomes, including higher cost and longer LOS. Additionally, the generalized linear regression indicated that the marginal mean cost difference between diabetic and non-diabetic patients was RMB (¥) 775.7.

**Conclusions:**

AECOPD patients with comorbid diabetes had a more severe profile and higher direct hospitalization cost. Diabetes screening and integrated care programs might help reduce the heavy comorbidity and economic burden. Moreover, corticosteroids and metformin could be considered in the treatment of these patients.

*Trial registration* Clinicaltrials.gov with the identifier NCT0265752.

## Introduction

Chronic obstructive pulmonary disease (COPD), the estimated third leading cause of death in the world by 2030 [1], imposes substantial comorbidity burden and economic burden [2, 3]. The comorbidity burden is higher for patients with two or more severe acute exacerbations of COPD (AECOPD) within three months than those without [4]. Similarly, diabetes occurs more frequently among COPD patients with greater than two exacerbation-caused readmissions compared to those with zero to one readmission [5]. Furthermore, AECOPD accounts for the greatest COPD-related burden on the healthcare system [6], and more than 70% of all COPD-related medical care cost was due to hospitalization [7].

Diabetes is one of common comorbidities among COPD patients [4, 8]. Comorbid diabetes has been reported to be associated with poor clinical outcomes, including increased hospitalization cost [9], increased length of stay (LOS) [10], and higher mortality [10–15] among COPD patients hospitalized with chronic bronchitis [9], patients without a main diagnosis of diabetes [10], patients with stroke [11, 13], heart failure [12], intracerebral hemorrhage [14], and patients with ovarian cancer [15]. However, among COPD patients hospitalized with exacerbation, it has been only researched for the association between diabetes or hyperglycemia and LOS and the association between diabetes and death [16, 17]. Furthermore, these studies are either retrospective or have small sample sizes. It has been rarely investigated for the impact of diabetes on healthcare cost among AECOPD patients. Diabetes was also analyzed as a covariate instead of a main analytic factor [18]. Additionally, even among COPD patients, the impact of diabetes on cost is controversial. Both positive [19] and negative [20] association have been reported.

To our knowledge, no prospective multicenter study has researched the relationship between diabetes and clinical outcomes among patients hospitalized with AECOPD and even the number of retrospective studies is limited. Thus, this study aimed to compare basic characteristics of AECOPD patients with and without comorbid diabetes, and to detect the association between diabetes and clinical outcomes, including direct hospitalization cost, LOS, and in-hospital death.

## Method

### Study design and patients

Acute exacerbation of Chronic obstrUctive pulmonary disease using REgistry data (ACURE) is an ongoing multicenter prospective patient registry study aiming to investigate the demographic characteristics, clinical features, diagnoses and treatments, prognoses, and economic cost among AECOPD patients. It planned to recruit 7600 COPD patients hospitalized due to exacerbation in real world settings from diverse areas of China. It started to enroll patients from 1st September 2017 and follow-up is expected to end in December 2022. Details of the ACURE cohort has been published elsewhere [21].

**Fig. 1:**
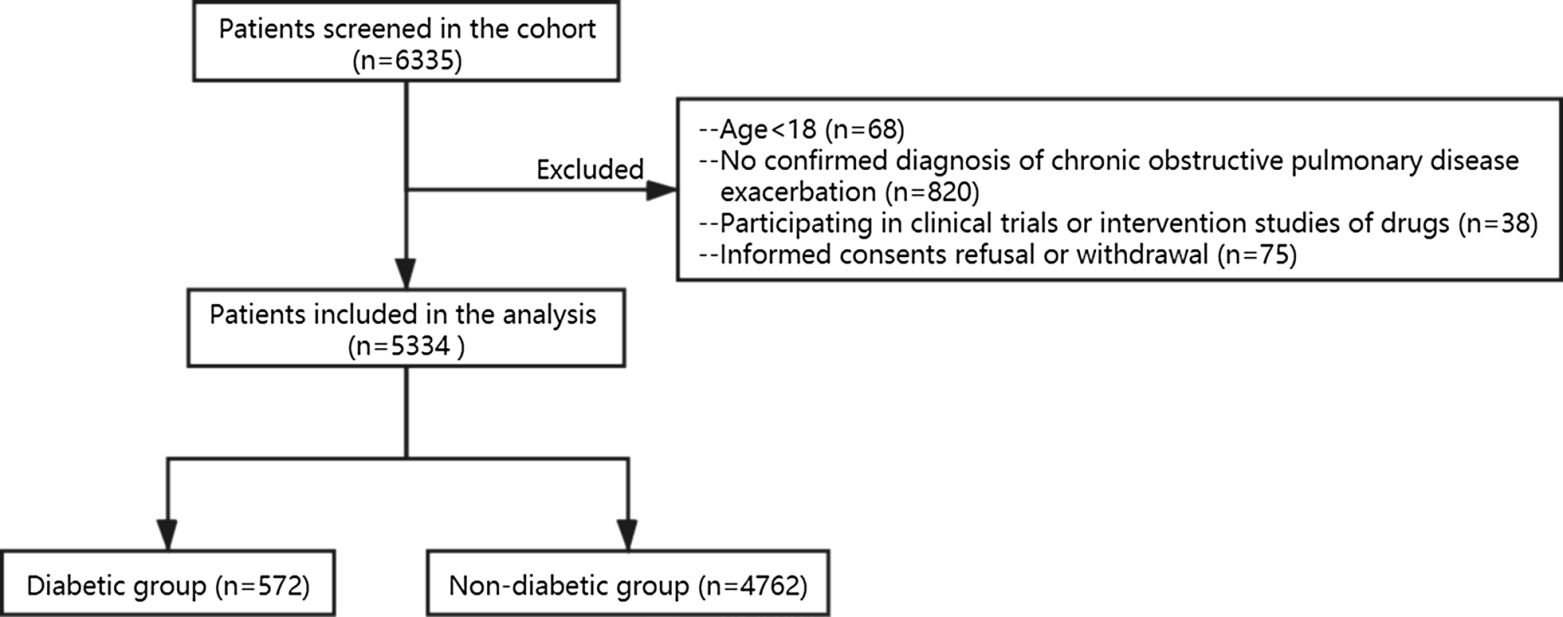
The flowchart of patient enrollment

Eligibility criteria of this study were: (1) 18 years or older; (2) inpatients with confirmed diagnosis of AECOPD; (3) available diabetes status. Patients were excluded if they withdrew the informed consent, had active pulmonary tuberculosis, or had acute left heart failure. The diagnosis of AECOPD was confirmed based on subjects’ personal history, clinical symptoms, and lung function level or former diagnosis of COPD. Some patients did not take lung function test during hospitalization due to the difficulty to take such tests during the period of exacerbation. For these patients, former test results or results within 30-day after discharge were used if available. Considering diabetes, the diagnosis was in accordance with the WHO 1999 diagnostic criteria or former diagnosis of diabetes. Baseline data on February 25th, 2020 (Phase I) was extracted for further analysis. Initially, 6335 patients were screened in the ACURE study. After exclusion, a total of 5334 patients from 163 centers were left for the analysis. Details are shown in Fig. 1.

### Outcome

- Primary outcomes: Direct hospitalization cost and LOS. Direct hospitalization cost was collected on the day of discharge and it includes diagnostic related fee (laboratory, pathology, inspection, etc.), medication, hospital stay, and other treatment related fee. LOS was defined as the duration between admission and discharge or death. It was classified into two groups: ≤ 10 days and > 10 days. 
- Secondary outcomes: In-hospital mortality.

### Variable definition

- Age was classified into categories: Age < 60, 60 ≤ age < 70, 70 ≤ age < 80, age ≥ 80 years
- Body mass index (BMI): BMI less than 18.5 kg/m^2^ was grouped as underweight, 18.5–23.9 kg/m^2^ was grouped as normal weight, 24–27.9 kg/m^2^ was grouped as overweight, and BMI more than 28 kg/m^2^ was classified as obesity [22].
- Global Initiative for Chronic Obstructive Lung Disease (GOLD) stage: According to the 2017 GOLD report, for patients with post-bronchodilator forced expiratory volume in 1s/forced vital capacity < 0.7 (FEV1/FVC < 0.7), it was categorized into four stages based on their post-bronchodilator FEV1% of predicted: Stage 1 (FEV1% ≥ 80), Stage 2 (50 ≤ FEV1% < 80), Stage 3 (30 ≤ FEV1% < 50), and Stage 4 (FEV1% < 30).
- COPD assessment test (CAT) was classified into four groups: 0–10, 11–20, 21–30, and 31–40.
- Cardiovascular comorbidities included coronary heart disease, heart failure, hypertension, etc.
- Respiratory comorbidities included asthma, pneumonia, lung cancer, etc.
- Digestive comorbidities included gastroesophageal reflux disease, cirrhosis, and peptic ulcers.

### Statistical analysis

Frequencies and proportions were calculated for categorical data, means and standard deviation (SD) or medians and interquartile range (IQR) were calculated for continuous variables. Characteristics of patients with and without diabetes were compared using χ^2^ test for categorical data. T-test or Wilcoxon rank-sum test was used for continuous data.

Regarding regression models, statistically significant factors in univariate analyses or clinically significant factors were considered for model selections. A generalized linear regression with a gamma distribution and a LOG link function was utilized to detect the association between diabetes and direct hospitalization cost due to its right skewness. Winsorization was utilized at 1% and 99% percentile of cost to prevent the impact of extreme cost outliers on the results. Diabetes, age group, sex, smoking, hospital level, intensive care unit or respiratory intensive care unit (ICU/RICU) admission, disease duration, insurance type, COPD assessment test (CAT) score at admission, corticosteroids use, cardiovascular comorbidities, respiratory comorbidities, digestive comorbidities, osteoporosis, and LOS (≤ 10 days and > 10 days) were included in the final model. Moreover, marginal difference for mean cost was reported for diabetes and covariates included in the final model. A multi-variable logistic regression model was utilized to analyze the association between diabetes and LOS (≤ 10 days and > 10 days). Diabetes, smoking, frequency of hospitalization due to AECOPD in the past 12 months, insurance type, cardiovascular comorbidities, respiratory comorbidities, digestive comorbidities, and CAT score at admission were included in the final model. All analyses were carried out using SAS 9.4 (SAS Institute Inc, Cary, North Carolina).

## Results

A total of 5334 AECOPD patients were evaluated, with a large proportion of male (78.8%) and a median (IQR) age of 69.0 (63.0, 76.0) years. Among these patients, 572 (10.72%) had comorbid diabetes. Diabetic patients were older, higher educated, more likely to be of overweight or obese, and had more former smokers but less current smokers. Moreover, diabetic patients had more emergency room visits in the past 12 months. Additionally, diabetic patients had a higher percentage of medical insurance for urban workers but lower percentage of medical insurance for urban and rural residents. For comorbidities, diabetic patients had more cardiovascular comorbidities (73.8% vs. 52.7%, P < 0.001), hypertension (55.8% vs. 31.2%, P < 0.001), asthma (12.4% vs. 7.9%, P < 0.001), bronchiectasis (16.1% vs. 12.2%, P = 0.007), and pneumonia (34.3% vs. 29.1%, P = 0.010); however, they had less pulmonary artery hypertension (6.8% vs. 9.1%, P = 0.070). Details are shown in Table 1.

**Table 1:** Characteristics of patients hospitalized with AECOPD: Overall and by diabetes status

	Total (N = 5334)	Diabetes (N = 572)	No diabetes (N = 4762)	P value
*Baseline characteristics*				
Age				0.008
N (missing)	5321 (13)	570 (2)	4751 (11)	
Median (IQR)	69.0 (63.0, 76.0)	70.0 (64.0, 77.0)	69.0 (63.0, 76.0)	
Age group				0.081
N (missing)	5321 (13)	570 (2)	4751 (11)	
Age < 60	769 (14.5)	63 (11.1)	706 (14.9)	
60 ≤ Age < 70	1956 (36.8)	209 (36.7)	1747 (36.8)	
70 ≤ Age < 80	1820 (34.2)	208 (36.5)	1612 (33.9)	
Age ≥ 80	776 (14.6)	90 (15.8)	686 (14.4)	
Sex, N (%)				0.787
N (missing)	5334 (0)	572 (0)	4762 (0)	
Female	1133 (21.2)	119 (20.8)	1014 (21.3)	
Male	4201 (78.8)	453 (79.2)	3748 (78.7)	
BMI, N (%)				< 0.001
N (missing)	5321 (13)	570 (2)	4751 (11)	
Underweight	886 (16.7)	51 (8.9)	835 (17.6)	
Optimal	2944 (55.3)	263 (46.1)	2681 (56.4)	
Overweight	1172 (22.0)	180 (31.6)	992 (20.9)	
Obesity	319 (6.0)	76 (13.3)	243 (5.1)	
Education level, N (%)				0.014
N (missing)	5334 (0)	572 (0)	4762 (0)	
Primary school and below	2591 (48.6)	245 (42.8)	2346 (49.3)	
High school	2520 (47.2)	300 (52.4)	2220 (46.6)	
College and above	223 (4.2)	27 (4.7)	196 (4.1)	
Smoking, N (%)				0.012
N (missing)	5334 (0)	572 (0)	4762 (0)	
Non-smoker	1715 (32.2)	186 (32.5)	1529 (32.1)	
Current smoker	1259 (23.6)	108 (18.9)	1151 (24.2)	
Former smoker	2360 (44.2)	278 (48.6)	2082 (43.7)	
*Past and current hospitalization*				
Frequency of hospitalization due to AECOPD in the past 12 months				0.053
N (missing)	5334 (0)	572 (0)	4762 (0)	
< 2	3785 (71.0)	386 (67.5)	3399 (71.4)	
≥ 2	1549 (29.0)	186 (32.5)	1363 (28.6)	
Frequency of emergency room visits due to AECOPD in the past 12 months				0.009
N (missing)	5327 (7)	571 (1)	4756 (6)	
< 2	4032 (75.7)	407 (71.3)	3625 (76.2)	
≥ 2	1295 (24.3)	164 (28.7)	1131 (23.8)	
Hospital level, N (%)				0.126
N (missing)	5334 (0)	572 (0)	4762 (0)	
Secondary	626 (11.7)	56 (9.8)	570 (12.0)	
Tertiary	4708 (88.3)	516 (90.2)	4192 (88.0)	
ICU/RICU admission, N (%)				0.268
N (missing)	5334 (0)	572 (0)	4762 (0)	
Yes	83 (1.6)	12 (2.1)	71 (1.5)	
No	5251 (98.4)	560 (97.9)	4691 (98.5)	
Disease duration of COPD (years), N (%)				0.374
N (missing)	5272 (62)	566 (6)	4706 (56)	
< 3	2310 (43.8)	235 (41.5)	2075 (44.1)	
3–5	1185 (22.5)	126 (22.3)	1059 (22.5)	
> 5	1777 (33.7)	205 (36.2)	1572 (33.4)	
Insurance type, N (%)				< 0.001
N (missing)	5334 (0)	572 (0)	4762 (0)	
No insurance	114 (2.1)	14 (2.4)	100 (2.1)	
Medical insurance for urban and rural residents	2804 (52.6)	239 (41.8)	2565 (53.9)	
Medical insurance for urban workers	2059 (38.6)	280 (49.0)	1779 (37.4)	
Other insurance	357 (6.7)	39 (6.8)	318 (6.7)	
Blood gases				
N (missing)	2338 (2996)	239 (333)	2099 (2663)	
PaO_2_ (mmHg), Median (IQR)	69.5 (59.7, 80.0)	70.0 (60.7, 80.0)	69.1 (59.2, 80.0)	0.482
PaCO_2_ (mmHg), Median (IQR)	42.0 (37.0, 47.3)	41.6 (36.7, 47.3)	42.0 (37.1, 47.3)	0.775
PH, Median (IQR)	7.4 (7.4, 7.4)	7.4 (7.4, 7.4)	7.4 (7.4, 7.4)	0.773
*Severity and corticosteroids use*				
CAT score at admission, N (%)				0.025
N (missing)	5334 (0)	572 (0)	4762 (0)	
0–10	505 (9.5)	68 (11.9)	437 (9.2)	
11–20	2253 (42.2)	218 (38.1)	2035 (42.7)	
21–30	2220 (41.6)	238 (41.6)	1982 (41.6)	
31–40	356 (6.7)	48 (8.4)	308 (6.5)	
GOLD stage, N (%)				0.225
N (missing)	3882 (1452)	407 (165)	3475 (1287)	
Stage 1	189 (4.9)	12 (2.9)	177 (5.1)	
Stage 2	1176 (30.3)	120 (29.5)	1056 (30.4)	
Stage 3	1611 (41.5)	172 (42.3)	1439 (41.4)	
Stage 4	906 (23.3)	103 (25.3)	803 (23.1)	
Corticosteroids use, N (%)				0.175
N (missing)	5328 (6)	572 (0)	4756 (6)	
Yes	4196 (78.8)	463 (80.9)	3733 (78.5)	
No	1132 (21.2)	109 (19.1)	1023 (21.5)	
*Comorbidity, N (%)*				
N (missing)	5334 (0)	572 (0)	4762 (0)	
Cardiovascular diseases	2930 (54.9)	422 (73.8)	2508 (52.7)	< 0.001
Hypertension	1803 (33.8)	319 (55.8)	1484 (31.2)	< 0.001
Respiratory diseases	3592 (67.3)	395 (69.1)	3197 (67.1)	0.355
Asthma	447 (8.4)	71 (12.4)	376 (7.9)	< 0.001
Bronchiectasis	671 (12.6)	92 (16.1)	579 (12.2)	0.007
Lung cancer	70 (1.3)	6 (1.0)	64 (1.3)	0.558
Pneumonia	1581 (29.6)	196 (34.3)	1385 (29.1)	0.010
Pulmonary artery hypertension	472 (8.8)	39 (6.8)	433 (9.1)	0.070
Pulmonary thromboembolism	18 (0.3)	2 (0.3)	16 (0.3)	> 0.999
Digestive diseases	192 (3.6)	19 (3.3)	173 (3.6)	0.706
Gastroesophageal reflux disease	100 (1.9)	11 (1.9)	89 (1.9)	0.928
Osteoporosis	70 (1.3)	9 (1.6)	61 (1.3)	0.561

AECOPD: Exacerbation of chronic obstructive pulmonary disease; BMI: Body mass index; CAT: COPD assessment test; COPD: Chronic obstructive pulmonary disease; GOLD: Global Initiative for Chronic Obstructive Lung Disease; ICU: Intensive care unit; IQR: Interquartile range; RICU: Respiratory intensive care unit

As to clinical outcomes of interest, the median (IQR) of direct hospitalization cost was higher in diabetic patients [RMB (¥) 11,088.6 (8032.0, 15,545.2) vs. RMB (¥) 9672.5 (7045.1, 13,640.0)]. Similarly, diabetic patients had a higher occurrence of longer LOS (> 10 days: 50.4% vs. 45.1%, P = 0.017). However, there was no difference for in-hospital mortality between these two groups. Details are shown in Table 2.

**Table 2:** Clinical outcomes of patients hospitalized with AECOPD: Overall and by diabetes status

	Total (N = 5334)	Diabetes (N = 572)	No diabetes (N = 4762)	P value
Direct hospitalization cost				< 0.001
N (missing)	5315 (19)	570 (2)	4745 (17)	
Median (IQR) (RMB, ¥)	9827.5 (7088.1, 13,890.0)	11,088.6 (8032.0, 15,545.2)	9672.5 (7045.1, 13,640.0)	
Length of Stay, N (%)				0.017
N (missing)	5315 (19)	567 (5)	4748 (14)	
≤ 10	2886 (54.3)	281 (49.6)	2605 (54.9)	
> 10	2429 (45.7)	286 (50.4)	2143 (45.1)	
CAT score at discharge, N (%)				0.306
N (missing)	5334 (14)	572 (1)	4749 (13)	
0–10	2242 (42.1)	225 (39.4)	2017 (42.5)	
11–20	2527 (47.5)	284 (49.7)	2243 (47.2)	
21–30	522 (9.8)	61 (10.7)	461 (9.7)	
31–40	29 (0.5)	1 (0.2)	28 (0.6)	
In-hospital death, N (%)				0.327
N (missing)	5334 (0)	572 (0)	4762 (0)	
Yes	8 (0.1)	0 (0.0%)	8 (0.2)	
No	5326 (99.9)	572 (100.0)	4754 (99.8)	

AECOPD: Exacerbation of chronic obstructive pulmonary disease; CAT: COPD assessment test; IQR: Interquartile range

The generalized linear regression was conducted with a total of 5218 patients included. What is more, direct hospitalization cost was winsorized at 1% and 99% percentile [53 (0.99%) patients of each direction]. Results showed that the marginal mean cost difference for diabetic and non-diabetic groups was RMB (¥) 775.7. Details are shown in Table 3. Contractively, diabetes was not a statistically significant risk factor for longer LOS (> 10 days) in patients hospitalized with AECOPD. Details are shown in Table 4.

**Table 3:** The association between diabetes and direct hospitalization cost: Generalized linear regression

Variable	Marginal mean cost difference (RMB, ¥)	P value
Diabetes (Yes vs. No)	775.7 (275.7, 1275.8)	0.002
Age group		
< 60	Reference	
60 ≤ Age < 70	142.4 (− 307.9, 592.7)	0.535
70 ≤ Age < 80	94.1 (− 369.5, 557.7)	0.691
≥ 80	1044.3 (464.6, 1623.9)	< 0.001
Sex (Male vs. Female)	812.6 (409.4, 1215.9)	< 0.001
Smoking		
Non-smoker	Reference	
Current smoker	− 128.2 (− 561.1, 304.7)	0.562
Former smoker	342.9 (− 49.5, 735.2)	0.087
Hospital level (Tertiary vs. Secondary)	2709.9 (2337.2, 3082.6)	< 0.001
RICU/ICU Admission (Yes vs. No)	10,762.5 (8508.3, 13,016.7)	< 0.001
Disease duration of COPD (years)		
< 3	Reference	
3–5	324.9 (− 47.8, 697.5)	0.087
> 5	1057.2 (711.7, 1402.7)	< 0.001
Insurance type		
No insurance	Reference	
Medical insurance for urban and rural residents	− 1796.6 (− 2964.4, − 628.9)	0.003
Medical insurance for urban workers	− 2280.2 (− 3453.5, − 1106.8)	< 0.001
Other Insurance	− 444.5 (− 1754.2, 865.1)	0.506
CAT score at admission		
0–10	Reference	
11–20	145.4 (− 343.5, 634.3)	0.560
21–30	1311.6 (807.2, 1816.0)	< 0.001
31–40	1872.2 (1108.2, 2636.2)	< 0.001
Corticosteroids use (Yes vs. No)	1540.3 (1208.8, 1871.9)	< 0.001
Cardiovascular comorbidities (Yes vs. No)	668.2 (367.0, 969.5)	< 0.001
Respiratory comorbidities (Yes vs. No)	616.3 (307.8, 924.8)	< 0.001
Digestive comorbidities (Yes vs. No)	1073.4 (220.1, 1926.6)	0.014
Osteoporosis (Yes vs. No)	1906.9 (423.4, 3390.3)	0.012
Length of Stay (> 10 vs. ≤ 10)	5360.0 (5044.0, 5676.0)	< 0.001

CAT: COPD assessment test; COPD: Chronic obstructive pulmonary disease; GOLD: Global Initiative for Chronic Obstructive Lung Disease; ICU: Intensive care unit; RICU: Respiratory intensive care unit

**Table 4:** The association between diabetes and LOS: Multivariate logistic regression

Variable	OR (95% CI)	P value
Diabetes (Yes vs. No)	1.08 (0.90, 1.29)	0.409
Smoking		
Non-smoker	Reference	
Current smoker	0.93 (0.80, 1.08)	0.347
Former smoker	1.14 (1.00, 1.30)	0.043
Frequency of hospitalization due to AECOPD in past the 12 months (≥ 2 vs. < 2)	1.28 (1.13, 1.45)	< 0.001
Insurance		
No insurance	Reference	
Medical insurance for urban and rural residents	1.20 (0.81, 1.79)	0.366
Medical insurance for urban workers	1.66 (1.11, 2.48)	0.014
Other Insurance	1.11 (0.71, 1.73)	0.660
Cardiovascular comorbidities (Yes vs. No)	1.39 (1.24, 1.56)	< 0.001
Respiratory comorbidities (Yes vs. No)	1.64 (1.45, 1.85)	< 0.001
Digestive comorbidities (Yes vs. No)	1.51 (1.12, 2.04)	0.007
CAT score at admission		
0–10	Reference	
11–20	1.04 (0.85, 1.28)	0.672
21–30	1.66 (1.35, 2.03)	< 0.001
31–40	2.22 (1.67, 2.96)	< 0.001

AECOPD: Exacerbation of chronic obstructive pulmonary disease; CAT: COPD assessment test; LOS: Length of stay, it was classified into two groups: ≤ 10 days and > 10 days

## Discussion

Our study found that the prevalence of diabetes among AECOPD patients in this study was 10.72%. Compared to non-diabetic patients, diabetic patients in this study generally had a more severe profile, including being older, more overweight or obese, having more former smokers, more hospitalization due to AECOPD and more emergency room visits in the past 12 months, and having higher burden of common comorbidities. Diabetic AECOPD patients also had worse clinical outcomes, including more patients with longer LOS and higher direct hospitalization cost. A marginal mean cost difference of RMB (¥) 775.7 was observed between diabetic and non-diabetic patients in the generalized linear regression. However, diabetes was not a risk factor for longer LOS (> 10 days).

The prevalence of diabetes among AECOPD patients in this study was 10.72%, which is slightly lower than the estimated standardized prevalence, 10.9%, among Chinese adult population in 2013 [23]. The prevalence of diabetes in this study was underestimated due to the lack of screening programs for diabetes. Moreover, the prevalence of diabetes in this study is close to 12.3% reported by Ngo et al. [24], lower than 25.3% reported by Terzano et al. [25] and 26.3% reported by Perera et al. [26]. Except for prevalence underestimation, comparing to the study of Terzano et al., this is probably due to younger age of participants in our study. Moreover, the prevalence of diabetes of China in 2013 was lower than the United States of America [27], which partially explains why the rate is lower than Perera et al.’s study.

Regarding the basic profile, the results of Jimenez-Garcia et al. agree with our findings that diabetic patients had a relatively more severe profile except for education, although it was for patients with stable COPD [28]. In our study, diabetic patients tended to have higher education in the univariate analysis. Potential explanation could be patients with higher education were more aware of health conditions, which increase the possibility for diagnosis of comorbid conditions, including diabetes. Moreover, education was not statistically significant after adjusting for other covariates. As to outcomes of interest, agreeing with other findings, diabetic AECOPD patients had longer LOS [17]. Similar to LOS, our finding found diabetic patients had higher hospitalization cost, which might be caused by complex treatments that were related to patients’ severe profile [29]. Specifically, diabetic patients had higher burden of other comorbidities, which could also possibly worsen COPD outcomes. However, our findings indicate no difference for in-hospital mortality in these two groups, which contrasts with the previous finding that diabetic patients had a higher percentage of death than non-diabetics [17]. Specifically, that study did not clarify the type of death (short-term or long-term) utilized in the analysis and did not compared the characteristics of diabetic and non-diabetic patients. This limited us to explore possible reasons that can cause conflicted results between these two studies. Moreover, it is a single center cross-sectional study.

Regarding the association between diabetes and clinical outcomes, cost difference was observed between diabetic and non-diabetic patients hospitalized with AECOPD in this study. This adds the evidence for the findings that diabetes was associated with a higher cost among COPD patients [30] and AECOPD patients [18]. However, our study found that diabetes was not a predictor of having longer LOS (> 10 days), which contrasts with previous findings [17, 25, 31]. Specifically, the population of our study is different from Parappil et al.’s study, and it is a single center study and might has underpowered results [17]. Considering the study of Wang et al. [31], diabetes was not the main analytic factor and COPD could be both the main diagnosis and secondary diagnosis, which had respiratory failure or pneumonia as the main diagnosis. It probably could also be explained that diabetes was not an independent predictor and was explained by other covariates, including insurance and frequency of hospitalization due to AECOPD in the past 12 months.

As discussed above, diabetic AECOPD patients had a more severe profile and higher burden of several clinical outcomes. Thus, approaches to reduce such burden should be explored among these patients. Firstly, our study found a relatively low prevalence of diabetes among AECOPD patients, which suggests the importance of diabetes screening to identify diabetic AECOPD patients. This suggestion is consistent with 2020 GOLD report that recommends health workers to routinely look for comorbidities [6]. If healthcare resource was scarce, the target population of screening could be high risk patients, including smoking patients [32], patients with related comorbidities such as cardiovascular disease, and patients treated with high doses of inhaled corticosteroids (ICS) [33]. A simple strategy to help screen is to ask patients about their dysglycemia status [34]. Secondly, our studies found diabetic patients had longer LOS and higher hospitalization cost. Moreover, although in-hospital mortality rate was not significantly different between diabetic and non-diabetic group in our study, the mortality rate was reported to be higher among diabetic AECOPD patients in the study of Parappil et al. [17]. A few treatment strategies could possibly help enhance the outcomes: (1) corticosteroids should not be discouraged among diabetic AECOPD patients, which is supported by two studies. One is conducted by Davies et al., which found, compring to the placebo group, the corticosteroid treated group of non-acidotic AECOPD patients requiring hospital admission had a shorter hospital stay [35]. Another one was done by Habib et al., which found corticosteroids was not associated with a significant increase in Hemoglobin A1C (HbA1c) among diabetic AECOPD patients [36]. (2) Metformin could be considered in the treatment for diabetic COPD patients since it may reduce death rate of these patients due to its pleiotropic anti-inflammatory and antioxidant effect [37]. As essential as screening and treatment, integrated care, including self-management and pulmonary rehabilitation, would help manage diabetic AECOPD patients better and thus might reduce the hospitalization cost [32]. (1) Self-management: Evidence has shown that exacerbation action plan, a component of self-management, could benefit COPD patients with comorbidities by decreasing duration per COPD exacerbation [38]. However, action plan [6] and education, another component of self-management, were not sufficient to achieve robust effect. However, by integrating them into a comprehensive integrated disease management program, mortality was reduced among COPD patients with history of exacerbation [39] or emergency room visit [39, 40] in the past year. (2) Pulmonary rehabilitation: It was reported that implementation after discharge following an AECOPD could reduce hospital admissions and improves quality of life or increases exercise capacity; however, further related research should investigate the proper time (how many weeks after discharge) for implementing such programs and whether the findings are consistent among diabetic AECOPD patients [41]. To conclude, the management of diabetic AECOPD patients, screening should be routinely administered. Concerning treatment, corticosteroids should not be discouraged, and metformin could be considered. Last but not least, comprehensive integrated care programs could be tried to check whether diabetic AECOPD patients had improving outcomes.

This study has several limitations. Firstly, the completion rate of lung function data is relatively low due to the difficulty to conduct such tests for patients with exacerbation during hospital stay. However, these patients were formerly diagnosed and had typical symptoms of exacerbation. Moreover, diabetes screening was not routinely done among all enrolled patients. However, to the best of our knowledge, our study is the first prospective multicenter registry study researching the association between comorbid diabetes and direct hospitalization cost and the association between diabetes and LOS among AECOPD patients. Additionally, for the association between comorbid diabetes and direct hospitalization cost, diabetes was firstly utilized as a main analytic factor, instead of a covariate.

This study suggests future research directions. It indicates the potential needs of diabetes screening programs among AECOPD patients to identify patients with higher burden and to get the prevalence of diabetes among AECOPD patients. Future studies, including evaluating causes of high hospitalization cost among diabetic AECOPD patients, are also needed to confirm our findings. Moreover, gaps of the effect of integrated care programs among AECOPD patients should be fulfilled.

## Conclusions

In conclusion, diabetic patients had a more severe profile, and it increased the direct hospitalization cost of patients hospitalized with AECOPD. Further studies with diabetes screening programs among AECOPD patients are useful to identify diabetic AECOPD patients and to confirm our findings, including risk factors of high hospitalization cost. Furthermore, integrated care programs could be utilized to verify the effects of such programs among diabetic AECOPD patients. Moreover, in the treatment of these patients, corticosteroids and metformin could be considered.

## Data Availability

The datasets analyzed during the current study are not publicly available but are available from the corresponding author on reasonable request.

## Abbreviations

ACURE: Acute exacerbation of Chronic obstrUctive pulmonary disease using REgistry data
AECOPD: Exacerbation of chronic obstructive pulmonary disease
BMI: Body mass index
CAT: COPD assessment test
COPD: Chronic obstructive pulmonary disease
FEV1: Forced expiratory volume in 1 s
FVC: Forced vital capacity
GOLD: Global Initiative for Chronic Obstructive Lung Disease
HbA1c: Hemoglobin A1C
ICS: Inhaled corticosteroids
ICU: Intensive care unit
IQR: Interquartile range
LOS: Length of stay
RCT: Randomized controlled trial
RICU: Respiratory intensive care unit
SD: Standard deviation
